# Supplementary material for: Macrophage ATP citrate lyase deficiency stabilizes atherosclerotic plaques
Source: Nat Commun. 2020 Dec 8;11:6296. doi: 10.1038/s41467-020-20141-z (PMC7722882; doi:10.1038/s41467-020-20141-z)
Supplement: Supplementary file 1 — Supplementary Information [file 41467_2020_20141_MOESM1_ESM.pdf]

Supplementary information for:

Macrophage ATP citrate lyase deficiency stabilizes atherosclerotic plaques

Jeroen Baardman[1,#], Sanne G.S. Verberk[2,#], Saskia van der Velden[1], Marion J.J. Gijbels[1,3], Cindy P.P.A. van Roomen[1], Judith C. Sluimer[3,4], Jelle Y. Broos[5,6], Guillermo R. Griffith[1], Koen H.M. Prange[1], Michel van Weeghel[7,8], Soufyan Lakbir[2,9], Douwe Molenaar[9], Elisa Meinster[2], Annette E. Neele[1], Gijs Kooij[5], Helga E. de Vries[5], Esther Lutgens[1,10], Kathryn E. Wellen[11], Menno P.J. de Winther[1,10] & Jan Van den Bossche[1,2]

[1]Department of Medical Biochemistry, Experimental Vascular Biology, Amsterdam Cardiovascular Sciences, Amsterdam UMC, University of Amsterdam, Amsterdam, Netherlands
[2]Department of Molecular Cell Biology and Immunology, Amsterdam Cardiovascular Sciences, Cancer Center Amsterdam, Amsterdam UMC, Vrije Universiteit Amsterdam, Amsterdam, Netherlands
[3]Department of Pathology and Molecular Genetics, CARIM, Maastricht University, Maastricht, Netherlands
[4] BHF Centre for Cardiovascular Sciences (CVS), University of Edinburgh, Edinburgh, UK
[5]Department of Molecular Cell Biology and Immunology, Amsterdam Neuroscience, MS Center Amsterdam, Amsterdam UMC, Vrije Universiteit Amsterdam, Amsterdam, Netherlands
[6] Leiden University Medical Center, Center for Proteomics & Metabolomics, Leiden, Netherlands
[7]Laboratory Genetic Metabolic Diseases, Amsterdam Cardiovascular sciences, Amsterdam UMC, University of Amsterdam, Amsterdam, Netherlands
[8]Core Facility Metabolomics, Amsterdam UMC, University of Amsterdam, Amsterdam, Netherlands
[9]Systems Bioinformatics, Vrije Universiteit Amsterdam, Amsterdam, Netherlands
[10]Institute for Cardiovascular Prevention (IPEK), Ludwig Maximilians University, Munich, Germany
[11]Department of Cancer Biology, Abramson Family Cancer Research Institute, Perelman School of Medicine, University of Pennsylvania, Philadelphia, USA
[#]These authors contributed equally.
email: j.vandenbossche@amsterdamumc.nl; m.dewinther@amsterdamumc.nl

Contents:

Supplementary Figure 1
Supplementary Figure 2
Supplementary Figure 3
Supplementary Table 1 - List of used antibodies
Supplementary Table 2 - List of primers used for the determination of gene expression levels
Supplementary Table 3 - Lipid characteristics for metabololipidomics with LC-MS

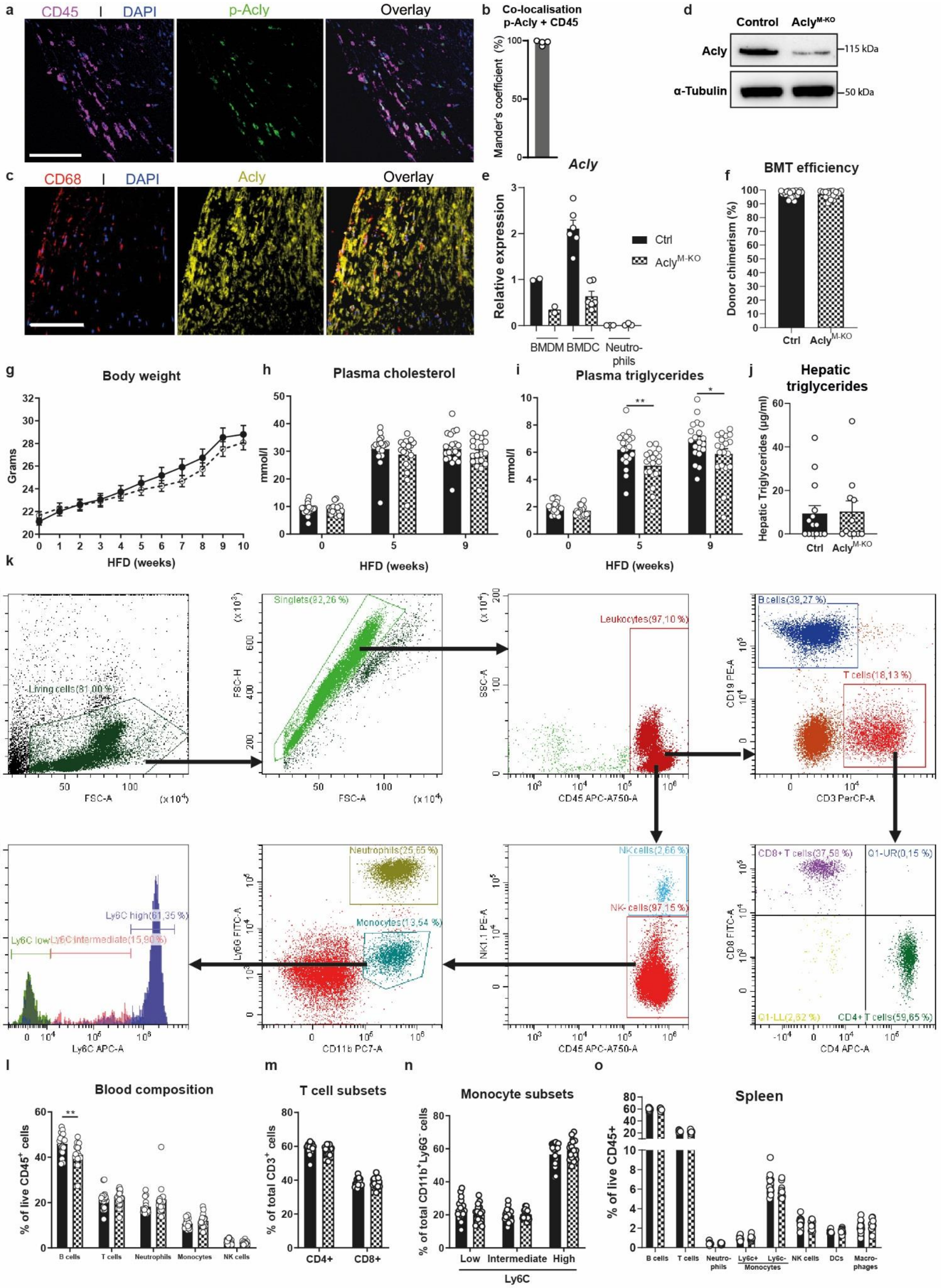

a
CD45 | DAPI
p-Acly
Overlay
b
Co-localisation p-Acly + CD45
Mander's coefficient (%)
d
Control
Acly M-KO
Acly
α-Tubulin
115 kDa
50 kDa
c
CD68 | DAPI
Acly
Overlay
e
Acly
Relative expression
Ctrl
Acly M-KO
BMDM
BMDC
Neutro-phils
f
BMT efficiency
Donor chimerism (%)
Ctrl
Acly M-KO
g
Body weight
Grams
HFD (weeks)
h
Plasma cholesterol
mmol/l
HFD (weeks)
i
Plasma triglycerides
mmol/l
HFD (weeks)
j
Hepatic triglycerides
Hepatic Triglycerides (µg/ml)
Ctrl
Acly M-KO
k
Living cells(81,00 %)
SSC-A
FSC-A
Singlets(92,26 %)
FSC-H
Leukocytes(97,10 %)
CD45 APC-A750-A
B cells(39,27 %)
T cells(18,13 %)
CD19 PE-A
CD3 PerCP-A
CD8+ T cells(37,58 %)
Q1-UR(0,15 %)
Q1-LL(2,62 %)
CD4+ T cells(59,65 %)
CD8 FITC-A
CD4 APC-A
NK cells(2,66 %)
NK- cells(97,15 %)
NK1.1 PE-A
Neutrophils(25,65 %)
Monocytes(13,54 %)
Ly6G FITC-A
CD11b PC7-A
Ly6C high(61,35 %)
Ly6C low
Ly6C intermediate(15,90 %)
Count
Ly6C APC-A
l
Blood composition
% of live CD45+ cells
B cells
T cells
Neutrophils
Monocytes
NK cells
m
T cell subsets
% of total CD3+ cells
CD4+
CD8+
n
Monocyte subsets
% of total CD11b+Ly6G- cells
Low
Intermediate
High
Ly6C
o
Spleen
% of live CD45+
B cells
T cells
Neutro-phils
Ly6c+
Ly6c-
Monocytes
NK cells
DCs
Macro-phages

Supplementary Fig. 1 (**a**) Representative immunohistochemical analysis for CD45 and p-Acly. Scale bar represents 100 µm. (n=4) (**b**) colocalization of CD45 and p-Acly. Scale bar represents 100 µm. (**c**) Representative immunohistochemical staining for CD68 and total Acly. (n=3 derived from surgery and autopsy. Staining pattern was similar in autopsy and surgery samples) (**d**) Protein levels of Acly as assed by immunoblotting (**e**) Relative gene expression of Acly in bone marrow derived macrophages, dendritic cells and neutrophils. (**f**) Donor chimerism. (**g**) Mouse weight during the course of the diet. (**h**) Plasma levels of cholesterol and (**i**) triglycerides before the start of the diet and five and nine weeks after the start of the diet **P=0.0077, *P=0.0342 (**j**) Hepatic triglyceride levels after 10 weeks of HFD (**k**) Gating strategy to asses composition of (**l, o**) leukocytes **P=0.0024, (**m**) T cell and (**n**) monocyte subsets in the blood and spleen, nine weeks after the start of the diet. (**o**) Spleen leukocyte composition after 10 weeks of HFD. Values represent mean ± SEM (n=4 (**b, e**) and n=20/19 (ctrl/KO in f-o)). *P < 0.05; **P < 0.01, ***P < 0.001 by two-way ANOVA with Bonferroni post hoc test for multiple comparisons (**i**) or by two-tailed Student's *t*-test (**l**). Source data are provided as a Source Data file (**b, d-j, l-o**)

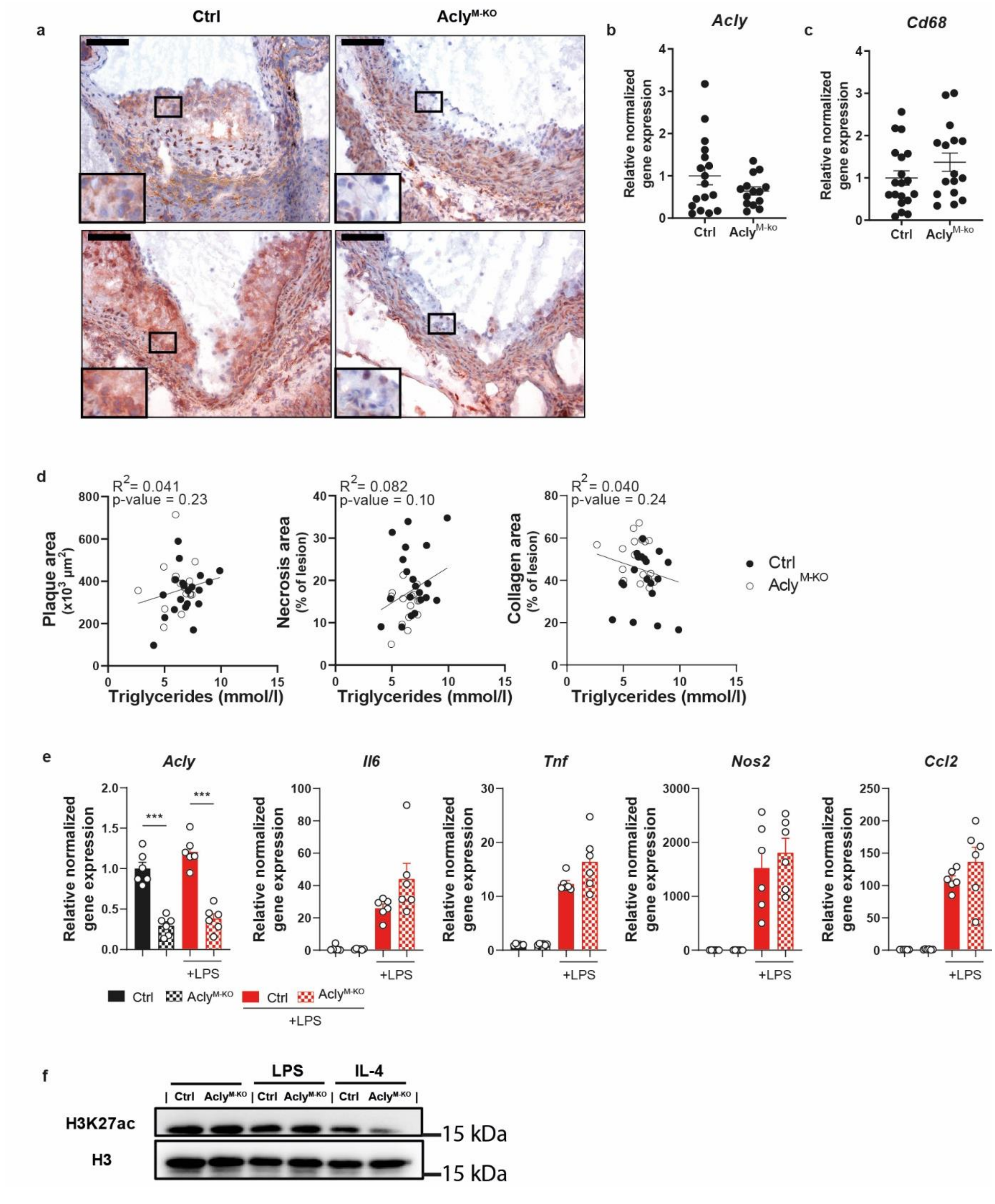

Supplementary Fig. 2 (**a**) Immunohistochemical staining of total Acly on lesions from. Scale bar represents 100 µm. (n=2/2 (Ctrl/KO))(**b**) Acly and (**c**) Cd68 expression in aortic arches (**d**) Correlations of plasma triglycerides with plaque phenotypes by linear regression. (**e**) *Acly* and inflammatory gene expression in untreated or 24 hours LPS-treated peritoneal cells from control and Acly$^{M\text{-}KO}$ *Ldlr*$^{-/-}$ mice. ***P<0.0001 (**f**) histone 3 K 27 acetylation (H3K27ac) in control and Acly$^{M\text{-}KO}$ medium, LPS or IL-4 treated macrophages. Representative image of 2 independent experiments. Values represent mean ± SEM n=20/19 (ctrl/KO in (**b,c**)) or n=6/6 (ctrl/KO in (**e**)) ***P < 0.001 by ordinary one-way ANOVA with Bonferroni post hoc test for multiple comparisons. R^2 and p-value data obtained by linear regression (**d**). Source data are provided as a Source Data file (**b-f**)

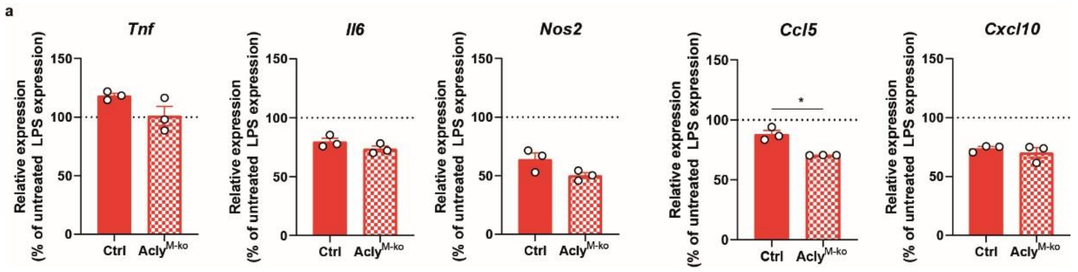

Supplementary Fig. 3 GW3965 affects control and Acly$^{M\text{-}KO}$macrophages (**a**) Inflammatory gene expression after GW3965 treatment relative to untreated LPS stimulated gene expression (=100%). Values represent mean ± SEM (n=4 technical replicates from 3 pooled mice) *P = 0.0051 by two-tailed Student's *t*-test. Source data are provided as a Source Data file

Supplementary table 1. List of used antibodies.

MARKER	CLONE	SUPPLIER	CATALOG NUMBER
**ACLY**	EP704Y	Abcam	ab40793
**PHOSPHORYLATED-ACLY (WB)**	Polyclonal	Cell Signaling Technology	4331
**A-TUBULIN**	B-5-1-2	Sigma-Aldrich	T5168
**HISTONE H3**	D2B12	Cell Signaling Technology	4620
**H3K27AC**	Polyclonal	Diagenode	C15410196
**ANTI-RABBIT IGG/HRP**	Polyclonal	Thermo Fisher Scientific	32260
**ANTI-MOUSE IGG/HRP**	Polyclonal	Thermo Fisher Scientific	32230
**CD16/CD32 (FC-BLOCK)**	93	eBioscience	14-0161
**CD71**	C2(F2)	BD Pharmingen	553267
**CD206**	C068C2	Biolegend	141707
**CD273**	TY25	BD Pharmingen	557796
**CD301**	ER-MP23	Serotec	MCA2392A647T
**IGG2A-PE (ISOTYPE CONTROL)**	RTK2758	Biolegend	400507
**IGG2A-APC (ISOTYPE CONTROL)**	RTK2758	Biolegend	400511
**CD45 (FACS)**	30-F11	BioLegend	103116
**CD11B**	M1/70	BD Pharmingen	552850
**LY6C**	ER-MP20	Serotec	MCA2389A647
**LY6G**	1A8	BD Pharmingen	551460
**NK1.1**	PK136	PK136	553165
**CD3**	145-2C11	eBioscience	45-0031
**CD4**	GK1.5	eBioscience	17-0041
**CD8**	53-6.7	eBioscience	11-0081
**CD19**	eBio1D3 (1D3)	eBioscience	12-0193
**MOMA-2**	MOMA-2	Serotec	MCA519G
**BIOTINYLATED RABBIT ANTI-RAT IGG ANTIBODY**	Polyclonal	Vector Laboratories	BA-4001
**PHOSPHORYLATED-ACLY (IHC)**	Polyclonal	Sigma	SAB4504020
**CD68**	KP1	Abcam	ab955
**TGF-B**	Polyclonal	Abcam	Ab92486
**CD45 (IHC)**	2B11+PD7/26	DAKO	M0701
**CD40**	3/23	Biolegend	124612
**MHCII**	M5/114.15	Biolegend	107626
**CD80**	16-10A1	Biolegend	104731
**CD86**	GL-1	Biolegend	105040
**MAC3**	M3/84	BD Pharmingen	550292
**ALEXA555 GOAT ANTI RAT**	Polyclonal	Molecular Probes	A21434
**ALEXA647 GOAT ANTI RABBIT**	Polyclonal	Molecular Probes	A27040
**ALEXA488 GOAT ANTI RABBIT**	Polyclonal	Molecular Probes	A11008
**ALEXA647 GOAT ANTI MOUSE**	Polyclonal	Invitrogen	A21237

Supplementary table 2. List of primers used for the determination of gene expression levels.

GENE	FORWARD PRIMER SEQUENCE	REVERSE PRIMER SEQUENCE
Ppia	TTCCTCCTTTCACAGAATTATTCCA	CCGCCAGTGCCATTATGG
Rplp0	GGACCCGAGAAGACCTCCTT	GCACATCACTCAGAATTTCAATGG
Acly	CCCCAAGATTCAGTCCCAAGT	GCCTTGGTATGTCGGCTGAA
Tgfb1	GTCACTGGAGTTGTACGGCA	AGCCCTGTATTCCGTCTCCT
Il6	GCTACCAAACTGGATATAATCAGGAAA	CTTGTTATCTTTTAAGTTGTTCTTCATGTACTC
Tnf	CATCTTCTCAAAATTCGAGTGACAA	TGGGAGTAGACAAGGTACAACCC
Nos2	GCAAACCCAAGGTCTACGTTCA	CCTCATTGGCCAGCTGCTT
Ccl2	AGCACCAGCCAACTCTCACT	CGTTAACTGCATCTGGCTGA
Il1b	AAAGAATCTATACCTGTCCTGTGTAATGAAA	GGTATTGCTTGGGATCCACACT
Arg1	CATGGGCAACCTGTGTCCTT	CGATGTCTTTGGCAGATATGCA
Retnla	CTGCCCTGCTGGGATGAC	TCCACTCTGGATCTCCCAAGA
Chil3	TGGCCCACCAGGAAAGTACA	CAGTGGCTCCTTCATTCAGAAA
Mgl2	GACAAAGGTTTCAAGAATTGGAGG	ACCTAGCATATGTCCATGCCA
Cd68	CGGCTCCCTGTGTGTCTGAT	CGTAGGGCTGGCTGTGCTT
Ccl5	GGAGTATTTCTACACCAGCAGCAA	GCGGTTCCTTCGAGTGACA
Cxcl10	CCAAGTGCTGCCGTCATTTT	AGCTTCCCTATGGCCCTCAT

Supplementary table 3. Lipid characteristics for metabololipidomics with LC-MS

GROUP	COMPOUND	LIPID MAPS ID	RETEN TION TIME [MIN]	M/Z IN Q1	M/Z IN Q3	DE-CLUST-ERING POTEN TIAL [V]	COLLI-SION ENERGY [V]	COLLI-SION CELL EXIT POT. [V]
**INTERNAL STANDARDS**	15-HETE-d8	LMFA03060080	7.8	327.2	226	-85	-18	-11
**INTERNAL STANDARDS**	DHA-d5	LMFA01030762	8.8	332	288.1	-75	-16	-13
**INTERNAL STANDARDS**	LTB4-d4	LMFA03020030	6.9	339.1	196.9	-70	-22	-19
**INTERNAL STANDARDS**	PGE2-d4	LMFA03010008	4.9	355.1	193	-50	-26	-17
**POLYUNSATURATED FATTY ACIDS**	AA	LMFA01030001	8.8	303	205.1	-155	-20	-11
**POLYUNSATURATED FATTY ACIDS**	AdA	LMFA01030178	9.1	331.1	233	-130	-22	-11
**POLYUNSATURATED FATTY ACIDS**	ALA*	LMFA01030152	8.6	277	182.1	-55	-24	-7
**POLYUNSATURATED FATTY ACIDS**	ALA/GLA	LMFA01030152 / LMFA01030141	8.6	277	233	-90	-22	-29
**POLYUNSATURATED FATTY ACIDS**	DGLA	LMFA01030158	9.0	305.1	261.2	-85	-22	-13
**POLYUNSATURATED FATTY ACIDS**	DHA	LMFA01030185	8.8	327.1	229.2	-115	-18	-11
**POLYUNSATURATED FATTY ACIDS**	DPAn-3	LMFA04000044	8.9	329.1	231.1	-50	-20	-17
**POLYUNSATURATED FATTY ACIDS**	DPAn-6	LMFA01030182	9.0	329.1	231.1	-50	-20	-17
**POLYUNSATURATED FATTY ACIDS**	EPA	LMFA01030759	8.6	301	202.9	-125	-18	-21
**POLYUNSATURATED FATTY ACIDS**	LA	LMFA01030120	8.8	279	261	-115	-28	-13
**PROSTAGLANDINS (PG)**	13,14-dihydro-15-keto-PGF2alpha	LMFA03010027	5.4	353.1	195	-110	-32	-11
**PROSTAGLANDINS (PG)**	15-deoxy-PGJ2	LMFA03010021	7.3	315	203	-50	-28	-19
**PROSTAGLANDINS (PG)**	15-keto-PGE2	LMFA03010030	4.5	349	234.9	-65	-20	-13
**PROSTAGLANDINS (PG)**	8-iso-PGE2	LMFA03110003	4.6	351.1	271	-5	-24	-19
**PROSTAGLANDINS (PG)**	8-iso-PGF2alpha	LMFA03110001	4.5	353.1	193	-135	-34	-11
**PROSTAGLANDINS (PG)**	PGD2	LMFA03010004	5.0	351.1	233	-30	-16	-13
**PROSTAGLANDINS (PG)**	PGE2	LMFA03010003	4.9	351.2	271.1	-50	-22	-21
**PROSTAGLANDINS (PG)**	PGF2alpha	LMFA03010002	5.2	353.1	193	-80	-34	-11
**PROSTAGLANDINS (PG)**	PGJ2	LMFA03010019	6.1	333	271	-30	-22	-17
