# Supplementary material for: Macrophage ATP citrate lyase deficiency stabilizes atherosclerotic plaques
Source: Nat Commun. 2020 Dec 8;11:6296. doi: 10.1038/s41467-020-20141-z (PMC7722882; doi:10.1038/s41467-020-20141-z)
Supplement: Supplementary file 3 — Description of Additional Supplementary Files [file 41467_2020_20141_MOESM3_ESM.docx]

Additional information supplementary data

File name: Supplementary Data 1

Description: Raw metabolipidomic measurements, relative RT and Area Ratio for indicated lipids in untreated and LPS-treated Ctrl and Acly^M-KO^ bone marrow derived macrophages.
