# Supplementary material for: Macrophage ATP citrate lyase deficiency stabilizes atherosclerotic plaques
Source: Nat Commun. 2020 Dec 8;11:6296. doi: 10.1038/s41467-020-20141-z (PMC7722882; doi:10.1038/s41467-020-20141-z)
Supplement: Supplementary file 5 — Reporting Summary [file 41467_2020_20141_MOESM5_ESM.pdf]

nature research

Corresponding author(s): Jan Van den Bossche

Last updated by author(s): Oct 5, 2020

Reporting Summary

Nature Research wishes to improve the reproducibility of the work that we publish. This form provides structure for consistency and transparency in reporting. For further information on Nature Research policies, see our Editorial Policies and the Editorial Policy Checklist.

Statistics

For all statistical analyses, confirm that the following items are present in the figure legend, table legend, main text, or Methods section.

n/a	Confirmed	
☐	☒	The exact sample size (n) for each experimental group/condition, given as a discrete number and unit of measurement
☐	☒	A statement on whether measurements were taken from distinct samples or whether the same sample was measured repeatedly
☐	☒	The statistical test(s) used AND whether they are one- or two-sided<br>*Only common tests should be described solely by name; describe more complex techniques in the Methods section.*
☒	☐	A description of all covariates tested
☐	☒	A description of any assumptions or corrections, such as tests of normality and adjustment for multiple comparisons
☐	☒	A full description of the statistical parameters including central tendency (e.g. means) or other basic estimates (e.g. regression coefficient) AND variation (e.g. standard deviation) or associated estimates of uncertainty (e.g. confidence intervals)
☐	☒	For null hypothesis testing, the test statistic (e.g. F, t, r) with confidence intervals, effect sizes, degrees of freedom and P value noted<br>*Give P values as exact values whenever suitable.*
☒	☐	For Bayesian analysis, information on the choice of priors and Markov chain Monte Carlo settings
☒	☐	For hierarchical and complex designs, identification of the appropriate level for tests and full reporting of outcomes
☐	☒	Estimates of effect sizes (e.g. Cohen's d, Pearson's r), indicating how they were calculated

Our web collection on statistics for biologists contains articles on many of the points above.

Software and code

Policy information about availability of computer code

Data collection	Data from RNA-sequencing were collected by aligning obtained reads to mouse genome mm10 by STAR (version 2.5.2b) with default settings. BAM files. were indexed and filtered on MAPQ>15 with SAMTools (version 1.3.1). RAW tag counts and RPKM values were summed using HOMER2's analyzeRepeats.pl script with default settings.<br>Raw liquid chromatography-mass spectrometry data was interpreted using Xcalibur software (version 4.1.31.9). Raw data from lipid HPLC-MS/MS were detected using the relative retention times, together with characteristic mass transitions specified in Supplementary Table 3.
Data analysis	Transcriptomic data were analyzed using the DESeq2 (version 1.24.0), ggplot2 (version 3.2), ggrepel (version 0.8.1) and pheatmap (version 1.0.12) packages in an R environment.<br>Metabolomics data were analyzed using the ggplot2 (version 3.2), ropls (version 1.18.8) and mixOmics (version 6.10.8) packages in an R environment.<br>Histological data were analyzed using Adobe Photoshop CS6 software (version 13.0.1).<br>Fluorescent images were analyzed using ImageJ vl.8.0_172 9 (version x) with a JACoP plugin.<br>Flow cytometry data was analyzed using CytExpert software (version 2.3.0.84).<br>Seahorse data was analyzed using Wave software (version 2.6.1).<br>Statistical analysis was performed using GraphPad Prism (version 8.2.1).

For manuscripts utilizing custom algorithms or software that are central to the research but not yet described in published literature, software must be made available to editors and reviewers. We strongly encourage code deposition in a community repository (e.g. GitHub). See the Nature Research guidelines for submitting code & software for further information.

Data

Policy information about availability of data

All manuscripts must include a data availability statement. This statement should provide the following information, where applicable:

- Accession codes, unique identifiers, or web links for publicly available datasets
- A list of figures that have associated raw data
- A description of any restrictions on data availability

All generated and analyzed data in this manuscript are available as a Source Data file. Any remaining data supporting the results of the study will be made available from the corresponding author upon reasonable request. RNA sequencing data is deposited in the GEO-database under accession number: GSE126690 (https://www.ncbi.nlm.nih.gov/geo/query/acc.cgi?acc=GSE126690). Raw data from lipidomics measurements are provided as Supplementary Data 1. Raw data from metabolomics measurements are deposited in MTBLS2159 (www.ebi.ac.uk/metabolights/MTBLS2159).

Field-specific reporting

Please select the one below that is the best fit for your research. If you are not sure, read the appropriate sections before making your selection.

☒ Life sciences ☐ Behavioural & social sciences ☐ Ecological, evolutionary & environmental sciences

For a reference copy of the document with all sections, see nature.com/documents/nr-reporting-summary-flat.pdf

Life sciences study design

All studies must disclose on these points even when the disclosure is negative.

Sample size	Sample size for the in vivo atherosclerosis study was determined as follows: Previous bone marrow transplantations (BMT) showed an average σ=47.5 and δ=45 for atherosclerotic plaque size. According to Sach's method, this leads to 17.5 animals per group. To account for possible drop-out of estimated maximum of 10%, group size should be 19.4 = 20 anmials. For 20 acceptors, 5 donor animals are necessary to provide sufficient material for transplantation. Therefore, each group consisted of 20 acceptants and 5 donors. This accounts for a total of 50 mice in this study, with 40 animals generating results.<br><br>In vitro studies were mostly performed on 3 technical replicates of bone marrow cells pooled from 3 mice, based on small variability seen in previous experiments. Representative data of one out of 2-5 individually performed experiments are shown.<br><br>For metabolomics, 6 technical replicates were used divided in 2 studies, since these experiments raise more variability. The average coefficient of variation in a biological replicate of a metabolomics measurement is ~20%. An effect of 35% can be considered relevant as was obtained from previous experiments . Based on a power analysis with 80% power and a unreliability threshold of 5% we determined that 6 biological replicates should be sufficient.
Data exclusions	Two AclyM-KO mice reached predetermined humane endpoint criteria during the study and were therefore not included in analysis.<br>Specific data was excluded based on ROUT's and Grubbs' methods for identifying outliers as determined by Graphpad Prism (version 8.0.2) or by analyzing whether data was outside the 1.5*interquartile range. In in vitro experimetns, outliers were only determined and excluded from analysis in sample sizes of n>6. Excluded data are available in the source data file with corresponding performed outlier test.
Replication	In vivo: The in vivo study was performed once under well-controlled circumstances in a sample size of 20.<br>Immunohistochemistry: Immunohistochemical stainings were performed once or twice with proper negative controls on tissue collected at different timepoints with differing plaque characteristics.<br>In vitro: In vitro experiments were replicated 2-4 times with similar results, results are shown of of one representative experiment.
Randomization	In vivo: Mice were allocated to 8 groups of 5 mice (4 Control groups and 4 AclyM-KO groups). Subsequently, the animals were randomly allocated to cages.<br>Immunohistochemistry: Immunohistochemical stainings and analyses were allocated into a random order.<br>In vitro: In in vitro experiments, mice were pooled and technical replicates were derived and tested under all conditions. Therefore, randomization is not relevant in these experiments.
Blinding	In vivo: Since injections for bone marrow transplantation were giving following randomization, the transplantation could not be performed blinded. This did not affect the efficiency of BMT as shown in the manuscript. All subsequent analyses were performed blinded and data was allocated into the correct groups after data analysis had finished.<br>Immunohistochemistry: Immunohistochemical stainings and analyses were performed blinded for plaque characteristics.<br>In vitro: In vitro experiments were not performed blinded

Reporting for specific materials, systems and methods

We require information from authors about some types of materials, experimental systems and methods used in many studies. Here, indicate whether each material, system or method listed is relevant to your study. If you are not sure if a list item applies to your research, read the appropriate section before selecting a response.

Materials & experimental systems

n/a	Involved in the study	
	x	Antibodies
	x	Eukaryotic cell lines
x		Palaeontology and archaeology
	x	Animals and other organisms
	x	Human research participants
x		Clinical data
x		Dual use research of concern

Methods

n/a	Involved in the study	
x		ChIP-seq
	x	Flow cytometry
x		MRI-based neuroimaging

Antibodies

Antibodies used

For each antibody listed:
MARKER-CLONE-SUPPLIER-CATALOG NUMBER
ACLY EP704Y Abcam ab40793
PHOSPHORYLATED-ACLY (WB) Polyclonal Cell Signaling Technology 4331
A-TUBULIN B-5-1-2 Sigma-Aldrich T5168
HISTONE H3 D2B12 Cell Signaling Technology 4620
H3K27AC Polyclonal Diagenode C15410196
ANTI-RABBIT IGG/HRP Polyclonal Thermo Fisher Scientific 32260
ANTI-MOUSE IGG/HRP Polyclonal Thermo Fisher Scientific 32230
CD16/CD32 (FC-BLOCK) 93 eBioscience 14-0161
CD71 C2(F2) BD Pharmingen 553267
CD206 C068C2 Biolegend 141707
CD273 TY25 BD Pharmingen 557796
CD301 ER-MP23 Serotec MCA2392A647T
IGG2A-PE (ISOTYPE CONTROL) RTK2758 Biolegend 400507
IGG2A-APC (ISOTYPE CONTROL) RTK2758 Biolegend 400511
CD45 (FACS) 30-F11 BioLegend 103116
CD11B M1/70 BD Pharmingen 552850
LY6C ER-MP20 Serotec MCA2389A647
LY6G 1A8 BD Pharmingen 551460
NK1.1 PK136 PK136 553165
CD3 145-2C11 eBioscience 45-0031
CD4 GK1.5 eBioscience 17-0041
CD8 53-6.7 eBioscience 11-0081
CD19 eBio1D3 (1D3) eBioscience 12-0193
MOMA-2 MOMA-2 Serotec MCA519G
LY6G 1A8 Pharmingen 551459
BIOTINYLATED RABBIT ANTI-RAT IGG ANTIBODY Polyclonal Vector Laboratories BA-4001
PHOSPHORYLATED-ACLY (IHC) Polyclonal Sigma SAB4504020
CD68 KP1 Abcam ab955
TGF-B Polyclonal Abcam Ab92486
CD45 (IHC) 2B11+PD7/26 DAKO M0701
CD40 3/23 Biolegend 124612
MHCII M5/114.15 Biolegend 107626
CD80 16-10A1 Biolegend 104731
CD86 GL-1 Biolegend 105040
MAC3 M3/84 BD Pharmingen 550292

Validation

All antibodies are validated according to manufacterer's product description as shown on their website as follows:
MARKER WEBSITE
ACLY https://www.abcam.com/atp-citrate-lyase-antibody-ep704y-ab40793.html
PHOSPHORYLATED-ACLY (WB) https://www.cellsignal.com/products/primary-antibodies/phospho-atp-citrate-lyase-ser455-antibody/4331?Ntk=Products&Ntt=4331
A-TUBULIN https://www.sigmaaldrich.com/catalog/product/sigma/t5168?lang=en&region=NL
HISTONE H3 https://www.cellsignal.com/products/primary-antibodies/histone-h3-d2b12-xp-rabbit-mab-chip-formulated/4620?Ntk=Products&Ntt=4620
H3K27AC https://www.diagenode.com/files/products/antibodies/Datasheet_H3K27ac_C15410196.pdf
ANTI-RABBIT IGG/HRP https://www.thermofisher.com/antibody/product/Goat-anti-Rabbit-IgG-H-L-Poly-HRP-Secondary-Antibody-Polyclonal/32260
ANTI-MOUSE IGG/HRP https://www.thermofisher.com/antibody/product/Goat-anti-Mouse-IgG-H-L-Poly-HRP-Secondary-Antibody-Polyclonal/32230
CD16/CD32 (FC-BLOCK) https://www.thermofisher.com/antibody/product/CD16-CD32-Antibody-clone-93-Monoclonal/14-0161-82

CD71 https://www.bdbiosciences.com/us/applications/research/stem-cell-research/mesenchymal-stem-cell-markers-bone-marrow/mouse/positive-markers/pe-rat-anti-mouse-cd71-c2-also-known-as-c2f2/p/553267
CD206 https://www.biolegend.com/en-us/products/apc-anti-mouse-cd206-mmr-antibody-7425
CD273 https://www.bdbiosciences.com/us/reagents/research/antibodies-buffers/immunology-reagents/anti-mouse-antibodies/cell-surface-antigens/pe-rat-anti-mouse-cd273-ty25/p/557796
CD301 https://www.bio-rad-antibodies.com/monoclonal/mouse-cd301-antibody-er-mp23-mca2392.html?f=alexa%20fluor%C2%AE%20647
IGG2A-PE (ISOTYPE CONTROL) https://www.biolegend.com/fr-lu/products/pe-rat-igg2a--kappa-isotype-ctrl-1843
IGG2A-APC (ISOTYPE CONTROL) https://www.biolegend.com/fr-lu/products/apc-rat-igg2a--kappa-isotype-ctrl-1838
CD45 (FACS) https://www.biolegend.com/fr-lu/products/apc-cyanine7-anti-mouse-cd45-antibody-2530
CD11B https://www.bdbiosciences.com/eu/applications/research/stem-cell-research/mesenchymal-stem-cell-markers-bone-marrow/mouse/negative-markers/pe-cy7-rat-anti-cd11b-m170/p/552850
LY6C https://www.bio-rad-antibodies.com/monoclonal/mouse-ly-6c-antibody-er-mp20-mca2389.html?f=alexa%20fluor%C2%AE%20647
LY6G https://www.bdbiosciences.com/eu/reagents/research/antibodies-buffers/immunology-reagents/anti-mouse-antibodies/cell-surface-antigens/fitc-rat-anti-mouse-ly-6g-1a8/p/551460
NK1.1 https://www.bdbiosciences.com/eu/reagents/research/antibodies-buffers/immunology-reagents/anti-mouse-antibodies/cell-surface-antigens/pe-mouse-anti-mouse-nk-11-pk136/p/553165
CD3 https://www.thermofisher.com/antibody/product/CD3e-Antibody-clone-145-2C11-Monoclonal/45-0031-82
CD4 https://www.thermofisher.com/antibody/product/CD4-Antibody-Monoclonal/17-0041-82
CD8 https://www.thermofisher.com/antibody/product/CD8a-Antibody-Monoclonal/11-0081-82
CD19 https://www.thermofisher.com/antibody/product/CD19-Antibody-Monoclonal/12-0193-82
MOMA-2 https://www.bio-rad-antibodies.com/monoclonal/mouse-macrophages-monocytes-antibody-moma-2-mca519.html?f=purified
BIOTINYLATED RABBIT ANTI-RAT IGG ANTIBODY https://vectorlabs.com/biotinylated-rabbit-anti-rat-igg-antibody-mouse-adsorbed.html
PHOSPHORYLATED-ACLY (IHC) https://www.sigmaaldrich.com/catalog/product/sigma/sab4504020?lang=en&region=NL
CD68 https://www.abcam.com/cd68-antibody-kp1-ab955.html
TGF-B https://www.abcam.com/tgf-beta-1-antibody-ab92486.html
CD45 (IHC) https://www.agilent.com/en/product/immunohistochemistry/antibodies-controls/primary-antibodies/cd45-leucocyte-common-antigen-(concentrate)-76507
CD40 https://www.biolegend.com/fr-lu/products/apc-anti-mouse-cd40-antibody-4984
MHCII https://www.biolegend.com/fr-lu/products/percp-cyanine5-5-anti-mouse-i-a-i-e-antibody-4282
CD80 https://www.biolegend.com/fr-lu/products/brilliant-violet-650-anti-mouse-cd80-antibody-7642
CD86 https://www.biolegend.com/fr-lu/products/brilliant-violet-510-anti-mouse-cd86-antibody-8745
MAC3 https://www.bdbiosciences.com/eu/reagents/research/antibodies-buffers/immunology-reagents/anti-mouse-antibodies/cell-surface-antigens/purified-rat-anti-mouse-cd107b-m384/p/550292
ALEXA555 GOAT ANTI RAT https://www.thermofisher.com/antibody/product/Goat-anti-Rat-IgG-H-L-Cross-Adsorbed-Secondary-Antibody-Polyclonal/A-21434
ALEXA647 GOAT ANTI RABBIT https://www.thermofisher.com/antibody/product/Rabbit-IgG-H-L-Secondary-Antibody-Oligoclonal/A27040
ALEXA488 GOAT ANTI RABBIT https://www.thermofisher.com/antibody/product/Goat-anti-Rabbit-IgG-H-L-Cross-Adsorbed-Secondary-Antibody-Polyclonal/A-11008
ALEXA647 GOAT ANTI MOUSE https://www.thermofisher.com/antibody/product/Goat-anti-Mouse-IgG-H-L-Cross-Adsorbed-Secondary-Antibody-Polyclonal/A-21237

Eukaryotic cell lines

Policy information about cell lines

Cell line source(s): Raw264.7 cells were originally obtained from ATCC

Authentication: RAW264.7 cells were authenticated based on their distinctive macrophage morphology and tested for the LPS-induced expression of know marker genes (Nos2, Tnf, Il6)

Mycoplasma contamination: The RAW264.7 cell line used in this study was tested negative on mycoplasma contamination by Lonza's MycoAlert protocol.

Commonly misidentified lines (See ICLAC register): No commonly misidentified lines were used in the study

Animals and other organisms

Policy information about studies involving animals; ARRIVE guidelines recommended for reporting animal research

Laboratory animals: C57Bl/6J mice with loxP sites flanking exon 9 of the Acly gene (Aclyfl/fl)14 were crossed with Lyz2-Cre transgenic mice to generate mice with a myeloid-specific deletion of Acly. Ldlr-/- mice on a C57BL/6 background were purchased from Jackson Laboratories. In vitro studies were performed on bone marrow from both male and female mice aged between 8-14 weeks. In vivo experiments were performed on female mice aged 8-10 weeks at the start of the study.

All mice were maintained in an animal facility at an ambient temperature of 20–24°C with 40–70% relative humidity under a 12:12 h

	light-dark cycle.
Wild animals	No wild animals were used in this study
Field-collected samples	No Field-collected samples were used in this study
Ethics oversight	All mouse experiments were conducted after approval by the Committee for Animal Welfare (University of Amsterdam and VU university Amsterdam).

Note that full information on the approval of the study protocol must also be provided in the manuscript.

Human research participants

Policy information about studies involving human research participants

Population characteristics	Formalin-Fixed Paraffin-Embedded (FFPE) human carotid samples collected at autopsy (n=10, mean age 72 years, all men) were used for immunohistochemistry.
Recruitment	From all consecutive donors from 1999-2010, a plaque was isolated and stored. Afterwards, a subselection was made based on plaque stadium and involved in this study. Past and current diagnosis and treatment did not play a role in selection of donors. Subselection bias does not play a role in the analysis and interpretation of the data.
Ethics oversight	The collection of study material was in line with the Dutch Code for Proper Secondary use of Human Tissue (https://english.ccmo.nl/investigators/types-of-research/non-wmo-research/research-with-human-tissue) and the Medical Ethical Committee AZm/UM from the Maastricht University Medical Centre. As the collection of these samples involved the secondary use of human tissue, this research is not subjective to research involving human subjects act and therefore not subjective to the declaration of Helsinki.

Note that full information on the approval of the study protocol must also be provided in the manuscript.

Flow Cytometry

Plots

Confirm that:

- [x] The axis labels state the marker and fluorochrome used (e.g. CD4-FITC).
- [x] The axis scales are clearly visible. Include numbers along axes only for bottom left plot of group (a 'group' is an analysis of identical markers).
- [x] All plots are contour plots with outliers or pseudocolor plots.
- [x] A numerical value for number of cells or percentage (with statistics) is provided.

Methodology

Sample preparation	To examine surface marker expression, BMDMs were detached using citrate buffer (17 mM tri-Sodium citrate dehydrate and 135 mM potassium chloride in water), transferred to V-bottom well plates, incubated with 1:50 anti-CD16/CD32 Fc-block and stained for 20 minutes at room temperature in the dark with antibodies.<br>Blood samples underwent red blood cell lysis using RBC lysis buffer from eBioscience. Spleen samples were digested with the use of an enzyme mix (Liberase TL). Blood an spleen were further processed similar to BMDM samples
Instrument	Beckman Coulter CytoFLEX S model B75442 and BD LXRFortessa X-20 cat. no. 65766M1 were used
Software	CytExpert was used to analyse data from Beckman Coulter CytoFLEX, and FlowJo was used to analyse data from BD LXR Fortessa
Cell population abundance	NA - Cells were not sorted during flow cytometry, only measured.
Gating strategy	In all experiments, cells ware gated from debris in a SSC-A/FSC-A gate and after negativety for a fixable viability dye. Blood and spleen samples were gated as follows: A CD45 gate for total leukocytes, Cd11b, MHCII, and Ly6C for monocytes, Ly6G for neutrophils, NK1.1 for natural killer cells, CD3, CD4, and CD8 for T cells and CD19 for B cells. Gates were set based positivity on a certain marker based on isotype controls.

- [x] Tick this box to confirm that a figure exemplifying the gating strategy is provided in the Supplementary Information.
